# Supplementary material for: On the asymptotic behavior of the average geodesic distance L and the compactness CB of simple connected undirected graphs whose order approaches infinity
Source: PLoS One. 2021 Nov 15;16(11):e0259776. doi: 10.1371/journal.pone.0259776 (PMC8592497; doi:10.1371/journal.pone.0259776)
Supplement: Attachment — Submitted filename: Response to Reviewers (PLOS ONE).pdf [file pone.0259776.s001.pdf]

# Response to Reviewers (PLOS ONE)

We kindly thank the reviewers for the useful comments and suggestions. Your remarks helped to a great extent to improve the article. In the following, we provide detailed answers to the questions.

## Reviewer 1

| **Reviewer comments** | **Response** |
|---|---|
| The paper provides proofs to two theorems that were stated (without proof) in another paper, published three years ago. I would like to remark that, although the manuscript has been submitted as a stand-alone paper, it would have been perhaps suitable as an appendix to the 2018 publication. A significant proportion of the preliminary materials from the 2018 publication are repeated in the current submission. | We would like to thank the reviewer for this comment. However, the results obtained here are independent from the previous paper and have their own contribution. In order to understand the paper without reading the previous one, we included the necessary definitions in the “Preliminaries”. |
| On a similar note, the authors claim in the “Concluding remarks” section that they will discuss the case of unconnected graphs in a follow-up paper. It is up to the editor to decide if the current results should be grouped with the future results into a more substantial publication or if instead, they should be separated as the authors propose. | Thank you for the suggestion. In the concluding remarks, we outlined our possible directions for future research. Considering your suggestion we decided to omit this outlook since at the moment we cannot estimate the possible scope of the future contribution. |
| In the introduction, the authors mention that the results provided in the manuscript are relevant to “answer questions regarding the structure of hypermedia”. Then, the authors move directly to the technical aspects of the paper. In my opinion, the paper would benefit from a more exhaustive introduction to help the reader understand the value and the context of the results. | We extended the introduction by providing details about the context of the present research. |
| Similarly, I would suggest to the authors to give a short review in the introduction that links their work with the existing literature on compactness. | Thank you for suggesting. We gave a review in the introduction. |

| | |
|---|---|
| Including the expression “of some graph classes” in the title seems vague given that the authors study only one particular class of graphs. A more concrete title would be: “About the possible limit values of compactness of simple connected undirected graphs”. | We completely agree with the reviewer that the original title of the paper is vague. We changed the title as suggested by the reviewer. Thank you for this suggestion. |
| In page 2, the authors give basic graph-theoretic definitions for the building-blocks of the paper. To remain coherent, they should also include what they mean by “simple graph”, “connected graph”, “sequence of graphs”, “isomorphic graphs”, “undirected graph”, “adjacent vertices”. | True. We included a reference where all these notions are explained in the first chapter. |
| On page 3, the authors introduce the mathematical definition of compactness. The intuition behind this concept can be deducted after reading the whole manuscript. However, the paper would be more readable if this definition was accompanied by the corresponding intuition of what this metric is measuring. | True. We provided a more detailed interpretation of compactness in particular with respect to the average geodesic distance in the discussion section. |
| Regarding the writing style, the authors use phrases as “obviously, …” or “one can easily see that …”. It is recommended to avoid these expressions as some statements may not be at all obvious for some readers (perhaps they only started researching graphs, or they may not have a mathematical background). | Thank you for this remark. To be precise, in this paper we expect some mathematical background to be present. In order to keep the text readable (without overloading it with detailed explanations of various evident facts) we use the expressions like “obviously, ..” etc. |

# Reviewer 2

| | |
|---|---|
| While the authors reference some relevant | The modified the Introduction section with respect to your suggestion. We hope our |

| | |
|---|---|
| bibliography, the wider context within which the article is situated is not successfully described. It would be desirable to provide the reader with clear information about what the contributions of the article are within the current state of affairs in the field. In particular, only one of the cited publications is from 2018 and is from the same author. The rest are at least from 2015 or older. In general, a broader perspective in terms of the offered references would be, to the reviewer criteria, needed. Along with this, it is recommended when the citations are made in the text, to provide the reader with the summarized information and the specific aspects for which the citation is made. | addition improves the overall understanding of the article. |
| The importance and relevance of the produced work should be highlighted, which links to point 3. | Done. |
| In general, while the structure of the text is suitable, the abstract, the introduction, as well as the conclusions sections would need some improvement.<br><br>Some suggestions are:<br><br>In the case of the abstract, to include the context and the relevance of the study. For the introduction, to present a wider context for the article, and probably leaving mathematical expressions for further in the text. Concluding remarks could also be improved including a summary of some of the information in the text, namely, the perspectives on future research offered; with a reference to relevance again; and to how the authors have contributed to the described current research context. | Following your suggestions, we changed the abstract, introduction, discussion and the "concluding remarks" sections, respectively. |
| Finally, although it seems that the authors have successfully built upon their work Lokot et al. 2018. The similarity of the title | Thank you for this suggestion, we changed the title to make it more precise. |

| | |
|---|---|
| and how it is referred to in the introduction do not help in making it clear. | |
| In line 126, implies that (instead of than). | True. We changed it accordingly. |
| In line 133, brackets are missing around 17 to be consistent with the rest of the text. | Thank you, we changed it. |
